# The comparative profile of lymphoid cells and the T and B cell spectratype of germ-free piglets infected with viruses SIV, PRRSV or PCV2

**DOI:** 10.1186/s13567-014-0091-x

**Published:** 2014-09-04

**Authors:** Marek Sinkora, John E Butler, Kelly M Lager, Hana Potockova, Jana Sinkorova

**Affiliations:** Laboratory of Gnotobiology, Institute of Microbiology of the Academy of Sciences of the Czech Republic, v.v.i., Novy Hradek, Czech Republic; Department of Microbiology, The University of Iowa, Iowa City, IA USA; Virus and Prion Research Unit, National Animal Disease Centre (NADC), USDA-ARS, Ames, IA USA

## Abstract

Lymphocyte subsets isolated from germ-free piglets experimentally infected with swine influenza virus (SIV), porcine reproductive and respiratory syndrome virus (PRRSV) or porcine circovirus type 2 (PCV2) were studied and the profile of these subsets among these three infections was monitored. Germ-free piglets were used since their response could be directly correlated to the viral infection. Because SIV infections are resolved even by colostrum-deprived neonates whereas PRRSV and PCV2 infections are not, SIV was used as a benchmark for an effectively resolved viral infection. PRRSV caused a large increase in the proportion of lymphocytes at the site of infection and rapid differentiation of B cells leading to a high level of Ig-producing cells but a severe reduction in CD2^—^CD21^+^ primed B cells. Unlike SIV and PCV2, PRRSV also caused an increase in terminally differentiated subset of CD2^+^CD8α^+^ γδ cells and polyclonal expansion of major Vβ families suggesting that non-specific helper T cells drive swift B cell activation. Distinct from infections with SIV and PRRSV, PCV2 infection led to the: (a) prevalence of MHC-II^+^ T cytotoxic cells, (b) restriction of the T helper compartment in the respiratory tract, (c) generation of a high proportion of FoxP3^+^ T cells in the blood and (d) selective expansion of IgA and IgE suggesting this virus elicits a mucosal immune response. Our findings suggest that PRRSV and PCV2 may negatively modulate the host immune system by different mechanisms which may explain their persistence.

**Electronic supplementary material:**

The online version of this article (doi:10.1186/s13567-014-0091-x) contains supplementary material, which is available to authorized users.

## Introduction

SIV, PRRSV and PCV2 are leading causes of disease in young pigs worldwide [1] and are responsible for significant economic losses with an estimated annual loss to PRRSV alone approaching 1 billion dollars just in the USA [2]. Vaccines are available for each of these viruses but they have variable efficacy. Currently all subunit vaccines for PRRSV have proven ineffective [H. Harris, Harris Vaccines, Ames, IA, personal communications]. Vaccines for PCV2 protect animals from clinical signs but the virus is not eliminated [3]. Limitation of vaccines against SIV that uses genetic reassortment is known [4]. Nevertheless, even germ-free (GF) piglets lacking passive antibodies (Abs) can resolve SIV infection within 6–7 days post challenge [5] whereas resolution of PRRSV [6,7] and PCV2 [8] infections is delayed. This delay may result from the ability to block, postpone or dysregulate an effective host immune response allowing the diseases to become pandemic. Since the mechanism of the successful resolution of SIV infection are well described [4] but no such information exist for delayed resolution of PRRSV and PCV2 infections, we wished to compare the lymphocyte profile of GF and SIV infected piglets with those infected with PRRSV and PCV2 in a setting in which only the virus can be responsible for the changes.

PRRSV is an enveloped, positive sense, single-stranded RNA virus with a 15.4 kb genome and it is divided into type 1 and type 2 genotypes based on European or North American origins, respectively [9]. Even though these genotypes emerged almost simultaneously and produce similar clinical signs, they share only about 70% identity at the nucleotide level [9]. Moreover, there are remarkable genetic variations among different PRRSV isolates within the same genotype, which is not surprising for an RNA virus. Clinical outcomes following PRRSV infection include respiratory disease, poor growth performance, increased mortality in young pigs and reproductive failure in sows [10]. The acute phase of viremia varies, usually covers ~28 days but can last beyond 50 days and in many cases, virus can be detected in lymph nodes for more than 100 days [10]. Pigs eventually develop sterilizing immunity although it may take months to become PCR negative. Thus there is a large window for spread to other animals and for in utero transmission of fatal disease to the fetus. PRRSV primarily targets monocyte/macrophage/dendritic lineage cells (Mo/MF/DC). Although infection with PRRSV induces a rapid and robust production of IgM followed by IgG [9,10], neutralizing Abs are slow to appear and their low titer makes them ineffective in clearance of the virus [10]. In fact, PRRSV viremia may be resolved without detectable levels of neutralizing Abs [11]. The appearance of IFN-γ secreting cells remains at a low level but slowly increases, plateauing at ~ 6 months postinfection. This T cell mediated response is ascribed mainly to effector/memory Th population with a minority of Tc cells [12].

PCV2 is a non-enveloped virus with a single-stranded circular DNA ~1.8 kb genome that is classified into genotype PCV2a and PCV2b displaying only minor antigenic differences [13]. However, PCV2 possesses the highest mutation rate reported for any DNA virus, falling into the range of genetic change reported for most RNA viruses [13]. PCV2 infects a wide range of cells including Mo/MF/DC, epithelia, endothelia and lymphocytes, and evidence suggests that virulence depends on the specific PCV2 isolate, regardless of genotype [13]. PCV2 is ubiquitous and many animals may be infected which may or may not result in clinical disease [1]. Porcine circovirus-associated disease encompasses a variety of clinical signs and diseases that are associated with PCV2 infection [14]. One of these, postweaning multisystemic wasting syndrome (PMWS), is a multifactorial disease complex which develops in weaned 5–12 week-old piglets [15]. It is characterized by weight loss, diarrhea, respiratory distress, enlarged lymph nodes and a generalized lymphopenia and loss of adaptive immunity [14–18]. Microscopic lesions consist of hepatitis, nephritis, damage of lymphoid tissue, and extensive lymphopenia [16–18]. Full expression of PMWS appears to require secondary factors such as co-infection, vaccination or other factors that activate the immune system [17–19]. Lymphopenia may predispose the host to immunosuppression leading to further complications in the form of co-infections with bacterial and viral pathogens. In subclinical infections Tc as well as Th cells are believed to play a protective role [18,20]. However, the response of T cells is generally weak [21]. Although the appearance of specific antibodies is delayed [16,21], subclinical PCV2 infection is characterized by the presence of neutralizing antibodies that correlate with protection from PCV2 infection [20].

In this study we compared the effect of these three viruses on the profile of lymphoid cell subsets in GF piglets. We adopted this strategy in the belief that the results obtained would represent the direct effect of the virus. Thus, the only environmental influence is through diet, since GF animals receive no maternal Abs or regulatory factors and are not colonized by normal gut microbiota [22–24]. Most important is that the effect of a viral infection on the immune response of GF piglets would not be masked as it is in conventional animals by effector and/or memory cells that are residual from previous exposure to microorganisms [24–27]. Also, GF piglets are currently the best model for fetal animals [7], which is especially relevant because PRRSV causes considerable fetal mortality [10] and PCV2 displays a high frequency of in utero transmission [3]. While the effect of these three viral infections on humoral response in GF piglets has been previously reported [5–7,28,29], the complex information about the phenotype of lymphoid cells and their distribution is missing. Here for the first time we report on the phenotypic profile of T, B and NK cells and their subsets utilizing our recent knowledge about these lymphoid cells in swine [24–27,30] allowing for comparative analysis of > 20 different lymphoid subpopulations. We wondered if the delay in resolution of PRRSV and PSV2 infections would be reflected in the lymphocyte profiles and if it might be a useful clue for explaining viral persistence but also help in the design of the next generation of vaccines for these pandemic diseases.

## Materials and methods

### Experimental design

In an effort to gain a better understanding of the effect of especially PRRSV and PCV2 on the piglet immune system, we conducted our studies in GF isolator piglets. Isolator piglets were selected since they can provide the best evidence for the direct effect of the virus on the neonatal immune system. In contrast to the hundreds of PRRSV studies done with conventional animals, we wished to use an experimental design that minimized the number of variables. Our extended strategy for future studies is to add to the GF model the environmental/maternal influences that would be encountered by conventional piglets. For example, subsequent studies could use gnotobiotic or caesarian-derived/colostrum-deprived (CDCD) piglets in which environmental factors could be introduced into the experiment.

Previously we reported on the humoral parameters of the same animals [5–7,28,29]. Based on those studies we were curious about how these infections influenced lymphocyte profiles. Since isolator piglets can only be maintained for 5–6 weeks and the number of animals is limited by costs, this study only provides kinetic data for the blood. Samples from all other tissues were only obtained at the termination of the study when the piglets were sacrificed. Majority of data are reported as relative levels of lymphocyte subsets, i.e. the proportion of each subset within given pool of cells. This parameter although not providing absolute numbers, might be more sensitive to indicate the direction being taken by the immune system when virally infected. We did calculate total blood cell numbers from leukogram data and compared these data to the relative/proportional data (see below). This comparison demonstrated that conclusions based on relative levels were similar to those reached when absolute levels were compared. In addition to collecting lymphocyte profile data, we also conducted spectratypic analyses, again with the goal of collecting preliminary data that could be used in the design of subsequent studies. In brief, our strategy was to gain a broad perspective on the comparative effects of three viral infections for the purpose of using the information obtained in the design of future hypothesis-driven studies.

### Experimental animals and infections

Pregnant gilts (Landrace × Yorkshire crossbred gilts bred to Duroc or Hampshire boars) from virus-free herds were anesthetized at the 112^th^ day of gestation and their fetuses were recovered by Caesarian under aseptic procedures [31]. A total of 68 piglets in three experiments were reared in sterile isolators on sterile formula as previously described [31,32]. Animals were distributed into isolators such that piglets in any one isolator were the offspring of two or more gilts. GF piglets were kept in isolator units under GF conditions at all times and monitored for bacterial contamination. Four days after birth (dpi 0) test animals were intranasally infected with a 1 mL dose of PRRSV (1 × 10^4^ CCID_50_, strain VR-2332, 8 animals) or SIV (1 × 10^4^ CCID_50_, isolate A/Swine/Iowa/15/1930 H1N1 subtype, 20 animals) or PCV2 (1 × 10^4^ CCID_50_, isolate 688, 28 animals). Twelve animals were maintained as GF controls. Some animals were immunized [31] intramuscularly with 6 mg of the T-independent antigen trinitrophenylated ficoll (TNP-Ficoll; 4 animals from the SIV group and 8 animals from the PCV2 group) or with 6 mg of the T-dependent antigen trinitrophenylated-keyhole limpet hemocyanin (TNP-KLH; 4 animals from the SIV group and 8 animals from the PCV2 group). Immunization was included because it was shown to promote the development of PMWS in PCV2 infected animals [19]. However, no differences were noted which is in agreement with observations of others [17,29]. Therefore, immunized and non-immunized animals were clustered and analyzed together. No immunization was used for PRRSV animals and therefore this group contained only 8 animals. Blood samples were collected prior to infection and thereafter weekly. All piglets were euthanized at dpi 28 and a spectrum of lymphoid tissues including blood was collected for flow cytometric studies or frozen in liquid nitrogen for later molecular biological studies. All animal studies were approved by the Institutional Animal Care and Use Committee (IACUC) of the National Animal Disease Center, USDA-ARS, Ames, Iowa, according to guidelines of the American Association for Laboratory Animal Care and the United States Department of Agriculture Animal Welfare Act.

### Leukogram

EDTA-treated blood samples of some animals were analyzed for white blood cell leukogram using a multispecies Hemavet HV950FS hematology system (Drew Scientific, Oxford, CT, USA).

### Preparation of cell suspensions

Cell suspensions were prepared essentially as previously described [33,34]. The same amount of tissue was examined for each animal and these samples were obtained and processed by the same procedures. Briefly, blood was obtained weekly by intravenous puncture of the jugular vein and by intracardial puncture at necropsy. Erythrocytes were removed using hypotonic lysis. Cell suspensions from the tracheobronchial lymph nodes (TBLN) and mesenteric lymph nodes (MLN) were prepared in PBS by teasing apart the tissues using a forceps and then by passage through a 70 μm mesh nylon membrane. Cells from the bronchoalveolar lavage (BAL) were recovered by washing lungs with PBS. Cell suspensions for flow cytometry were thereafter washed twice in PBS containing 0.1% sodium azide and 0.2% gelatin from Cold Water Fish Skin (PBS-GEL), filtered finally through a 70 μm mesh nylon membrane and cell numbers were determined by hemacytometer.

### Immunoreagents

The following mouse anti-pig monoclonal Abs, whose source and specificity were described earlier [34,35], were used as primary immunoreagents: anti-CD2 (1038H-5-37, IgM), anti-CD3 (PPT3, IgG1), anti-CD4 (10.2H2, IgG2b), anti-CD8α (76-2-11, IgG2a), anti-CD21 (IAH-CC51, IgG2b), anti-CD25 (K231-3B2, IgG1), anti-TCRγδ (PPT16, IgG2b), anti-IgM (M160, IgG1), anti-IgA (M1456, IgG2a) and anti-SLA-DR (swine MHC class II leukocyte Ag type DR (1038H-12-34, IgM). Forkhead box P3 protein (FoxP3) expression was detected by intracellular staining with biotinylated rat anti-mouse Ab FJK-16 s (eBioscience, San Diego, CA, USA). Goat polyclonal Abs specific for mouse immunoglobulin subclasses labeled with fluorescein isothiocyanate, R-phycoerythrin, Phycoerythrin/Cyanin 7 tandem complex or Allophycocyanin were used as secondary immunoreagents. Biotinylated monoclonal Abs were detected by a streptavidin-Phycoerythrin/Cyanin 7 conjugate (all secondary reagents from Southern Biotechnologies Associates, Inc., Birmingham, AL, USA). All immunoreagents were titrated for optimal signal/noise ratios. Primary isotype-matched mouse anti-rat monoclonal Abs were used as negative controls. Secondary polyclonal Abs were tested for cross-reactivity (no primary monoclonal Abs) and also for cross-reactivity with primary isotype-mismatched mouse anti-pig monoclonal Abs.

### Staining of cells and analysis of individual subpopulations

Staining of cells for flow cytometry analysis was performed as described previously [33,36,37] by indirect sub-isotype staining. Briefly, multi-color staining was done using cells that had been incubated with a combination of primary mouse monoclonal Abs of different sub-isotypes. Cells were incubated for 30 min and subsequently washed twice in PBS-GEL. Mixtures of goat secondary polyclonal Abs specific for mouse immunoglobulin subclasses that had been labeled with different fluorochromes were then added to the cell pellets in appropriate combinations. After 30 min, cells were washed three times in PBS-GEL and analyzed by flow cytometry. In the case of intracellular staining for FoxP3, cells that had been indirectly stained for cell surface molecules were subsequently intracellularly stained using IntraStain Kit according to a protocol recommended by the manufacturer (DakoCytomation, Glostrup, Denmark).

The gating strategy and analysis of individual subpopulations is depicted in Additional file 1. The frequency of lymphoid cells was determined as the proportion of cells in lymphoid gate (cells in R1) among all analyzed cells (A). The proportion of B cells was determined as the percentages of IgM^+^ cells from forward scatter versus side scatter (FSC/SSC) gated lymphocytes (cells in R2, B). The proportions of individual CD2/CD21 B cell subpopulations were detected from gated IgM^+^ lymphocytes (C). According to our recent studies [25,26,38] anti-CD21 Ab used in this study (IAH-CC51) stains CD21^b^ isoform that is expressed differentially on functionally distinct subsets of IgM^+^ mature B cells. Thus IgM^+^ B cells are composed of CD2^+^CD21^+^ naive B cells, CD2^—^CD21^+^ primed B cells, CD2^+^CD21^—^ effector antibody-forming cells and plasma cells (AFC/PC) and CD2^—^CD21^—^ resting AFC/PC. The proportions of IgM/IgA B cell subpopulations were detected from FSC/SSC gated lymphocytes (B). The proportions of αβ T and γδ T cells were determined as the percentages of CD3^+^TCRγδ^—^ (D, cells in R2) and CD3^+^TCRγδ^+^ (D, cells in R3) gated lymphocytes respectively. The proportions of individual CD2/CD8α γδ T cell subpopulations were determined from gated TCRγδ^+^ lymphocytes (E). The same procedure was used for detection of MHC-II^+^ γδ T cells (F). The proportions of individual CD4/CD8α αβ T cell subpopulations were determined as the percentages of CD4^—^CD8α^hi^ CTL (G, cells in R2), CD4^+^CD8α^—^ naive Th (G, cells in R4) and CD4^+^CD8α^+^ effector/memory Th (G, cells in R3) cells from FSC/SSC gated lymphocytes. Gating for these CD4/CD8α αβ T cell subpopulations allows the proportions of MHC-II^+^ cells within these subpopulations to be determined (H-J). Note that CD25 expression was not analyzed in this work (see Results). Analysis of αβ T regulatory cells was done by analysis of FoxP3/CD25 subpopulations from gated CD4^+^ lymphocytes (K). The proportion of NK cells was determined as the percentage of CD3^—^CD8α^+^ gated lymphocytes (L).

### Flow cytometry

Samples were measured on a FACSCalibur flow cytometer (BDIS, Mountain View, CA, USA). Electronic compensation was used to eliminate residual spectral overlaps between individual fluorochromes. PCLysis software (BDIS) was used for data processing. Data are reported as relative levels of lymphocyte subsets.

### PCR amplification and CDR3 spectratyping

The diversity in the B and αβ T cell repertoires is overwhelmingly determined by the diversity in CDR3 (IGHV-CDR3 and TCRBV-CDR3 respectively) [38–41]. Thus, length analyses of CDR3 regions using polyacrylamide sequencing gels provides a clonotypic analysis of porcine B cells [39–42] and αβ T cells [43] showing their level of clonality. Briefly, frozen tissue was dissolved in 0.5 mL TRI Reagent and total RNA was isolated according to a protocol recommended by the manufacturer (Sigma-Aldrich, St. Louis, MO, USA). Total cDNA was prepared using random hexamer primers. Each cDNA preparation was amplified in nine concurrent analyses (three for TCRBV-CDR3 spectratyping and six for IGHV-CDR3 spectratyping), each analysis consisted of three rounds of PCR. All primers used for amplifications are listed in Table 1. The 1^st^ round PCR targeted the original cDNA preparation while the 2^nd^ round PCR targeted the 1^st^ round PCR products. The CDR3 spectratyping was done in the 3^rd^ round PCR that involved incorporation of radioactive markers into the 2^nd^ round PCR products. Specifically, half of the α-Cβ2 primer (Table 1, primer No. 9) and the α-J_H_ primer (Table 1, primer No. 18) was ^32^P labeled and the products were separated on sequencing gels. Gels were dried and images were obtained indirectly by Kodak X-Omat Blue XB-1 film developed in X-Ray Processor. Profile curves were obtained by Carestream Molecular Imaging Software (Rochester, NY, USA).

**Table 1:** **List of primers used in this study**

**TCRBV amplifications** ^**1**^			**IGHV amplifications** ^**2**^		
**No.:**	**Primer:**	**Sequence:**	**No.:**	**Primer:**	**Sequence**
1	VβI	tccatgctcttctgctgtgt	10	FR1-5	gaggagaagctggtggagt
2	VβIV	kgcaycgggstkctctg	11	FR1-3	ctcctgtgtcggctctgga
3	VβVII	ctcascggramcctttgc	12	FR3	tgagaaccgaagacacggc
4	FR3βIa	gactchgchstgtwyytctgtg	13	α-Cμ	gggacgaagatgttcaagac
5	FR3βIb	crgacatctvkrtayytstgtg	14	α-Cδ	gctgggagctgccgagat
6	FR3βIV	gactcsgcygtgtatctctg	15	α-Cγ	ccgtccacgtaccaggagaa
7	FR3βVII	gactcrgccacctacstctg	16	α-Cα	gagcccaggagcaggtct
8	α-Cβ1	tctccgcttccgatggttca	17	α-Cε	gtccggatggtggtgtttg
9	α-Cβ2	gtggtctcacctgctgcag	18	α-J_H_	tgaggacacgacgacttcaa

^1^Following primer pairs (stated in brackets for 1^st^, 2^nd^ and 3^rd^ PCR respectively) were used for TCRBV amplifications: VβI-VβIII families (1/8, 1/9, 4 + 5/9), VβIV-VβVI families (2/8, 2/9, 6/9) and VβVII family (3/8, 3/9, 7/9). Annealing temperatures (Tm) were 54 °C for 1^st^ PCR, 61 °C for 2^nd^ PCR and 55 °C for 3^rd^ PCR.

^2^Following primer pairs (stated in brackets for 1^st^, 2^nd^ and 3^rd^ PCR respectively) were used for IGHV amplifications: total immunoglobulins (10/18, 11/18, 12/18), IgM (10/13, 11/13, 12/18), IgD (10/14, 11/14, 12/18), IgG (10/15, 11/15, 12/18), IgA (10/16, 11/16, 12/18) and IgE (10/17, 11/17, 12/18). Annealing temperatures (Tm) were 58 °C for 1^st^ and 2^nd^ PCR and 55 °C for 3^rd^ PCR.

### Statistical analysis

All data are proportional because they were acquired by flow cytometry from gated pools of specified cells. Most data are expressed individually (scatter plots) but in some cases, data are expressed as the mean ± standard deviation. Differences among the experimental values were analyzed by one way analysis of variance (ANOVA) – Bonferroni Multiple Comparison test or by paired t-test using GraphPad Prism4™ software (GraphPad Software, San Diego, CA, USA). The level of statistical significance is reported in *P*-values: *P* < 0.05 was considered significant, *P* < 0.01 was considered highly significant and *P* < 0.001 was considered very highly significant.

## Results

### Clinical and virological observations

This study involves monitoring the lymphoid cells profile in the same set of GF piglets that had been previously used in studies on humoral parameters including antibody repertoire development [5–7,29]. The clinical response of SIV, PRRSV, and PCV2 infected piglets during the course of these studies (dpi 0–28) is described therein. Briefly, SIV infected piglets were slightly affected post inoculation as recognized by a transitory decrease in appetite beginning dpi 3–4 and lasting for a few days. All SIV infected animals were no longer viremic dpi 7–10 and showed no further clinical signs [5]. At necropsy lung lesions were no longer present but the TBLN remained enlarged. PRRSV infected piglets were mildly affected based on a decrease in appetite dpi 3–4 which lasted for 7–14 days. Although the onset and duration was variable, mild dyspnea frequently developed at about dpi 7 and lasted 7–14 days. At dpi 28 mild pulmonary lesions were observed in some piglets and PRRSV was detected in all [7]. Subjectively, TBLN were larger compared to SIV infected piglets and there was also more systemic responses with increased size in MLN as well as other lymph nodes. The majority of PCV2 animals remained normal post inoculation but one PCV2 infected piglet developed PMWS and died on dpi 25 (this animal developed lymphopenia and was omitted from analyses; see the Discussion and Additional file 2). PCV2 infected animals did not resolve the infection by the end of experiments and all were PCR positive [29]. Subjective evaluation revealed that PCV2 infected piglets were similar to the PRRSV infected piglets as regards the size of lymph nodes, albeit TBLN were not as enlarged as with PRRSV but were larger than for SIV. All inoculated piglets in each experiment replicated the virus used for inoculation and were seropositive for respective virus while control piglets remained free of viral infection and remained asymptomatic [5–7,29]. Leukograms for individual infections (Additional file 3) show that there was no effect of SIV infection on total number of white blood cells, neutrophils or lymphocytes but the number of monocytes was increased. This agrees with findings of relative monocytosis observed during H1N1 virus infection [44]. On the other hand, PRRSV infection caused an increase in total white blood cells that was associated with an increase in neutrophils, lymphocytes and also monocytes (Additional file 3). This also agrees with findings of others because although the decrease in total number of white blood cells and lymphocytes was reported for PRRSV in very early stages after infection (dpi 3–7), subsequent increase of these parameters was also observed [45,46]. Infection with PCV2 caused a decrease in total white blood cells that was associated with a decrease in lymphocytes (Additional file 3). This also agrees with observations of others [17,21].

### SIV, PRRSV and PCV2 infections result in characteristic lymphocyte profiles at the site of infection

**Figure 1:**
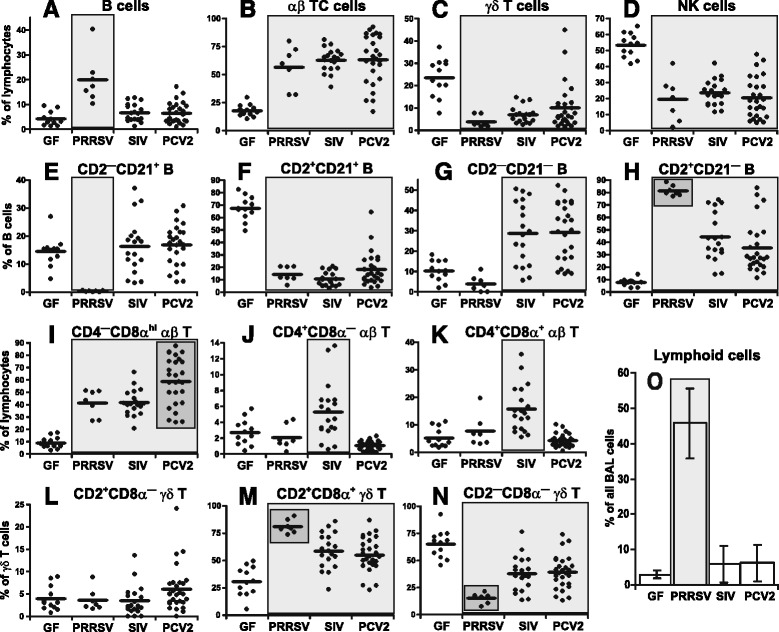
**Analysis of lymphoid cells and their subpopulations in the BAL.** Cell suspensions isolated from the BAL of control GF piglets and GF piglets infected with PRRSV, SIV or PCV2 (different animal groups depicted on x-axes) were analyzed by flow cytometry for the proportion of B cells **(A)** and their subpopulations **(E-H)**, αβ T cells **(B)** and their subpopulations **(I-K)**, γδ T cells **(C)** and their subpopulations **(L-N)** and NK cells **(D)**. The proportion of cells in lymphoid regions is also shown **(O)**. The phenotype of individual cell populations is depicted above each graph. Each dot in the graphs represents an individual animal and the mean for all animals in the same group is indicated by a horizontal line. Groups of animals that show a statistically significant difference (*p* < 0.05) from GF animals are light-grey boxed and those differing even within a group are dark-grey boxed.

At some point during infection, SIV, PRRSV and PCV2 impact the respiratory tract. Therefore, we analyzed the phenotype of cells in the bronchoalveolar lavage (BAL). Flow cytometry analysis revealed that while the proportion of IgM^+^ B cells was significantly increased only in the case of PRRSV infection (Figure 1A), all three viruses caused an increase in the proportion of αβ T cells (Figure 1B). The increase in the proportion of αβ T cells in all three infections was associated with a decrease in the proportion of γδ T cells (Figure 1C) and NK cells (Figure 1D).

When different subpopulations of IgM^+^ B cells were analyzed (Figure 1E-H), all three infections caused a substantial decrease in the proportion of naive CD2^+^CD21^+^ B cells compared to GF controls (Figure 1F). This decrease was associated with a corresponding increase in the proportion of effector CD2^+^CD21^—^ AFC/PC which was especially pronounced with PRRSV (Figure 1H) but also for resting CD2^—^CD21^—^ AFC/PC in SIV and PCV2 infections (Figure 1G). The substantial increase in effector CD2^+^CD21^—^ AFC/PC in PRRSV infection (Figure 1H) was accompanied by a severe reduction of primed CD2^—^CD21^+^ B cells (Figure 1E). In contrast, the proportion of this B cell subset in SIV and PCV2 infections was comparable to that in GF animals (Figure 1E).

Analysis of αβ T cell subsets (Figure 1I-K) revealed that all three infections caused a significant increase in CTL (CD4^—^CD8α^hi^ αβ T cells), which was highest for PCV2 infection (Figure 1I). On the other hand, only SIV infection produced an increase in naive CD4^+^CD8α^—^ (Figure 1J) and in effector/memory CD4^+^CD8α^+^ (Figure 1K) Th cells. Noteworthy was a very uniform reduction in the proportion of both of these Th subpopulations in PCV2 infected animals (Figure 1J and K).

The proportion of the CD2^+^CD8α^—^ γδ T cell subset was comparable to GF animals in all three infections (Figure 1L). However, the CD2^+^CD8α^+^ γδ T subpopulation was increased in all three viral infections with the greatest increase seen in PRRSV infection (Figure 1M). The opposite pattern was observed for the CD2^—^CD8α^—^ γδ T subpopulation in which relative levels were lower than in GF controls (Figure 1N).

It is notable that PRRSV infection caused a ~10-fold increase in the proportion of lymphoid cells in the BAL cells (Figure 1O). This is especially relevant because there was a remarkable increase in the proportion of B cells in the BAL (Figure 1A), namely effector CD2^+^CD21^—^ AFC/PC (Figure 1H). Therefore, these cells are ~40-fold more frequent in PRRSV infection when compared to SIV or PSV2 infections. Analysis of lymphoid cells (Figure 1O) also revealed that the contribution of non-lymphoid cells (such as macrophages, monocytes, neutrophils and others) in BAL of SIV or PCV2 infected piglets was the same as for GF animals.

### The draining lymphoid nodes for the lung show major differences in the T cell compartment

**Figure 2:**
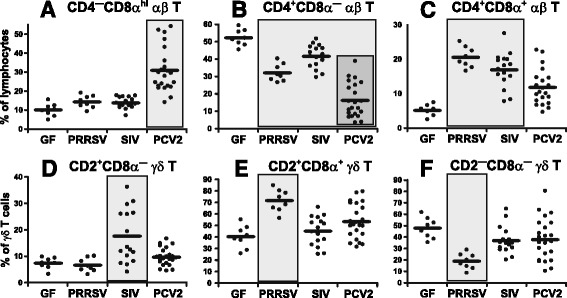
**Analysis of lymphoid cells and their subpopulations in the TBLN.** Cell suspensions isolated from TBLN of control GF piglets and GF piglets infected with PRRSV, SIV or PCV2 (different animal groups depicted on x-axes) were analyzed by flow cytometry for the proportion of αβ T cell subpopulations **(A-C)** and γδ T cell subpopulations **(D-F)**. The phenotype of individual cell subpopulations is depicted above each graph. Note that the proportion of B cells and their subpopulations and also the proportion of total αβ T, γδ T and NK cells did not differ between experimental groups and therefore these are not shown. Each dot in the graphs represents an individual animal and the mean for all animals in the same group is indicated by a horizontal line. Groups of animals that show a statistically significant difference (*p* < 0.05) from GF animals are light-grey boxed and those differing even within a group are dark-grey boxed.

The TBLN are the draining lymph nodes for the lungs. Analysis of TBLN cells for frequencies of all IgM^+^ B, αβ T, γδ T and NK cells did not reveal any significant differences between GF and infected animals (data not shown). The same applies for individual subpopulations of IgM^+^ B cells (data not shown). However, analysis of αβ T cell subpopulations (Figure 2A-C) showed a significantly higher proportion of CTL in PCV2 infection (Figure 2A), while PRRSV and SIV infections were characterized by a significantly higher occurrence of effector/memory Th cells (Figure 2C). The proportional contribution of naive Th cells was lower in all three infections than in GF controls (Figure 2B). The highest decrease was observed in PCV2 infections (Figure 2B).

The distributions of γδ T cell subpopulations in the TBLN also differed among various infections (Figure 2D-F). While SIV caused an increase in the proportion of cells in the CD2^+^CD8α^—^ subset (Figure 2D), PRRSV caused an increase in the CD2^+^CD8α^+^ subset (Figure 2E) resulting in a proportional decrease in the CD2^—^CD8α^—^ subset (Figure 2F). Infection with PCV2 does not affect the proportion of any γδ T cell subpopulation (Figure 2D-F).

### SIV, PRRSV and PCV2 differentially affect the proportions of lymphoid cells in the MLN

**Figure 3:**
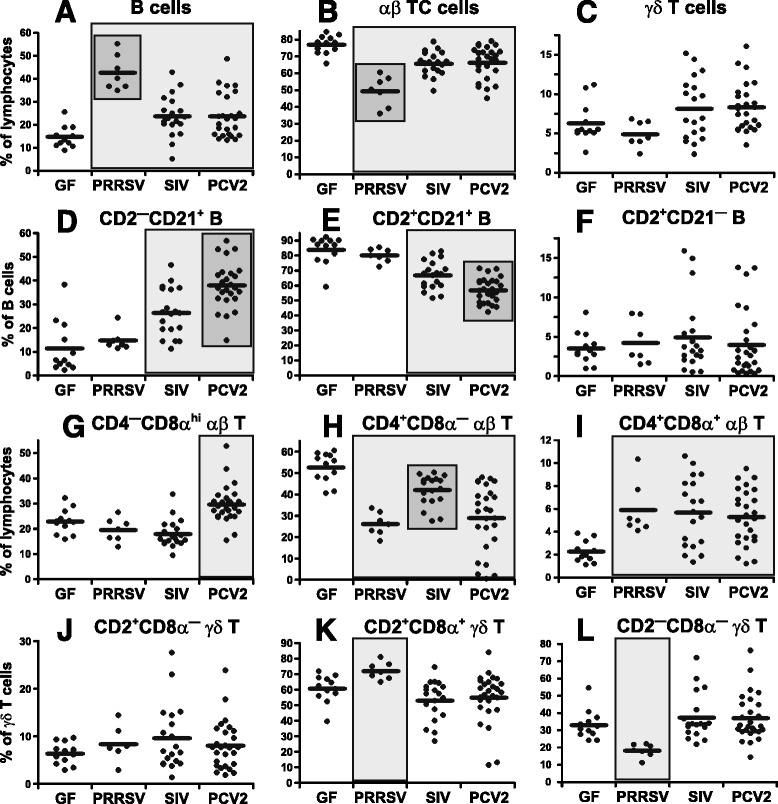
**Analysis of lymphoid cells and their subpopulations in the MLN.** Cell suspensions isolated from MLN of control GF piglets and GF piglets infected with PRRSV, SIV or PCV2 (different animal groups depicted on x-axes) were analyzed by flow cytometry for the proportion of B cells **(A)** and some of their subpopulations **(D-F)**, αβ T cells **(B)** and their subpopulations **(G-I)** and γδ T cells **(C)** and their subpopulations **(J-L)**. The phenotype of individual cell subpopulations is depicted above each graph. Note that the proportion of NK cells and CD2^—^CD21^—^ B cell subpopulation did not differ between experimental groups and therefore these are not shown. Each dot in the graphs represents an individual animal and the mean for all animals in the same group is indicated by a horizontal line. Groups of animals that show a statistically significant difference (*p* < 0.05) from GF animals are light-grey boxed and those differing even within a group are dark-grey boxed.

Analysis of lymphocytes in the MLN was done to examine whether a respiratory infection was reflected in the immune response in gut-associated lymphoid tissue. Figure 3 shows that the proportion of IgM^+^ B cell in the MLN was increased relative to GF controls in all three infections, the most obvious being in PRRSV infection (Figure 3A), which is similar to what was seen in the BAL (Figure 1A). This increase in B cells resulted in a proportionally lower level of αβ T cells (Figure 3B). There was no apparent impact of any infection on the frequencies of γδ T cells (Figure 3C) or NK cells (data not shown), which remained similar to that in GF controls.

Analysis of IgM^+^ B cell subpopulations in the MLN (Figure 3D-F) revealed a proportional increase in the primed CD2^—^CD21^+^ subset in SIV and PCV2 infections while the level in PRRSV infected piglets was similar to GF controls (Figure 3D). The opposite pattern was observed for naive CD2^+^CD21^+^ B cells (Figure 3E). These findings contrast with what was observed in the BAL where the proportion of primed CD2^—^CD21^+^ B cells in PRRSV infected animals dropped below the level seen in the BAL of GF controls (compare Figure 3D with Figure 1E). The proportions of effector CD2^+^CD21^—^ (Figure 3F) and resting CD2^—^CD21^—^ AFC/PC (data not shown) in the MLN remained unchanged in all three viral infections. This is predicted from previous observation that AFC/PC stimulated at remote sites do not home to the MLN but rather migrate to other gut-associated lymphoid tissues [26].

Similar to the BAL and TBLN, an increase in the proportion of CTL characterized PCV2 infection (Figure 3G). On the other hand, the proportion of effector/memory Th cells was increased in all three infections relative to GF piglets (Figure 3I) while naive Th cells were decreased (Figure 3H). This partially corresponds with the increase in effector/memory Th cells in the TBLN (compare Figure 3I with Figure 2C and Figure 3H with Figure 2B).

Analysis of γδ T cell subpopulations in the MLN (Figure 3J-L) revealed no significant change in the proportion of CD2^+^CD8α^—^ γδ T cells in any infection (Figure 3J). However, PRRSV infection caused an increase in CD2^+^CD8α^+^ (Figure 3K) but a decrease in the CD2^—^CD8α^—^ subset (Figure 3L), while infection with SIV and PCV2 had no effect (Figure 3J-L). This pattern was very similar to what was observed for lymphocytes in the TBLN (Figure 2D-F) and partially similar to what was seen in the BAL (Figure 1L-N).

### Changes in the proportions of lymphocyte subsets in the blood reflect only partially what was seen in the respiratory tract

Weekly analyses of the blood showed that the proportions of IgM^+^ B, αβ T, γδ T and NK cells did not change during the course of any infection and were comparable to GF animals (data not shown). However, when relative numbers were recalculated to absolute numbers according to leukogram data (Additional file 3), the total number of IgM^+^ B cells in the blood was increased in PRRSV but decreased in PCV2 infected animals compared to GF controls (Additional file 4). A similar pattern was seen for αβ T cells. There was no apparent impact of any infection on the absolute numbers of γδ T cells or NK cells, which remained similar to those in GF controls.

**Figure 4:**
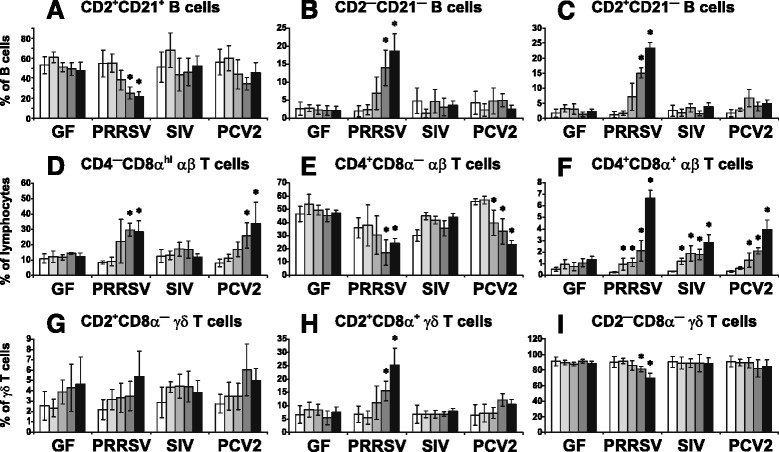
**Analysis of lymphoid cells and their subpopulations in the blood.** Cell suspensions isolated from the blood of control GF piglets and GF piglets infected with PRRSV, SIV or PCV2 (different animal groups depicted on x-axes) were analyzed by flow cytometry for the proportion of some B cell subpopulations **(A-C)**, αβ T cell subpopulations **(D-F)** and γδ T cell subpopulations **(G-I)**. The phenotype of individual cell subpopulations is depicted above each graph. Note that the proportion of total B, αβ T, γδ T and NK cells did not differ between experimental groups and therefore these are not shown. The same applies for CD2^—^CD21^+^ B cell subpopulation. Bars represent mean values obtained during monitoring of animals at dpi 0 (open bars), 7 (light-grey bars), 14 (dark-grey bars), 21 (light-black bars) and 28 (black bars). Error bars represent ± standard deviation. Values that show statistically significant difference (*p* < 0.05) from day 0 values are indicated by asterisk. Statistical analyses were done using paired t-test so that levels of significance reflect behavior of all pigs in a given group.

Analysis of IgM^+^ B cell subpopulations in terms of relative numbers revealed that the significant changes occurred only during PRRSV infection where the proportion of naive CD2^+^CD21^+^ B cells gradually decreased (Figure 4A), while the proportions of resting CD2^—^CD21^—^ AFC/PC (Figure 4B) and effector CD2^+^CD21^—^ AFC/PC cells (Figure 4C) remarkably increased. Interestingly, the restriction of primed CD2^—^CD21^+^ B cells observed in the BAL of PRRSV infected piglets (Figure 1E) was not observed in the blood where the proportion of this subset was comparable to GF controls during all three infections (data not shown). Comparison of relative numbers with recalculated absolute numbers (Additional file 4) showed similar changes with these exceptions (1) no difference in the absolute number of blood CD2^+^CD21^+^ B cells in PRRSV infected animals was seen while relative numbers decreased and (2) the decrease in the absolute number of CD2^+^CD21^+^ B cells in PCV2 infected animals was not significant when expressed in relative numbers.

As regards αβ T cell subpopulations, the proportion of effector/memory Th cells progressively increased in all infections compared to GF controls but was most pronounced in PRRSV infections (Figure 4F). The proportional increase in effector/memory Th cells at dpi 28 was very highly significant between PRRSV and SIV, and highly significant between PRRSV and PCV2 (Figure 4F). On the other hand, relative numbers for CTL were only elevated for PRRSV and PCV2 infected animals (Figure 4D). These relative increases occurred concomitantly with a relative decrease in naive Th cells in PRRSV and PCV2 infected animals (Figure 4E). Comparison of relative numbers with recalculated absolute numbers (Additional file 4) showed again similar changes with these exceptions (1) an increase in CTL in PCV2 infected animals, which is significant in the case of relative numbers is not significant in the case of absolute numbers and (2) a decrease in naive Th cells in PRRSV infected animals, which is significant in the case of relative numbers is not significant in the case of absolute numbers.

Changes in the proportion of circulating γδ T cell subsets only occurred during PRRSV infection. Similar to other analyzed tissues, the proportion of CD2^+^CD8α^+^ subset increased (Figure 4H), while the proportion of CD2^—^CD8α^—^ subset decreased (Figure 4I). This agrees with recalculated absolute numbers (Additional file 4) with exception that a decrease in relative numbers of CD2^—^CD8α^—^ subset cannot not be observed.

### MHC-II expression on T cells occurs in all three viral infections

**Figure 5:**
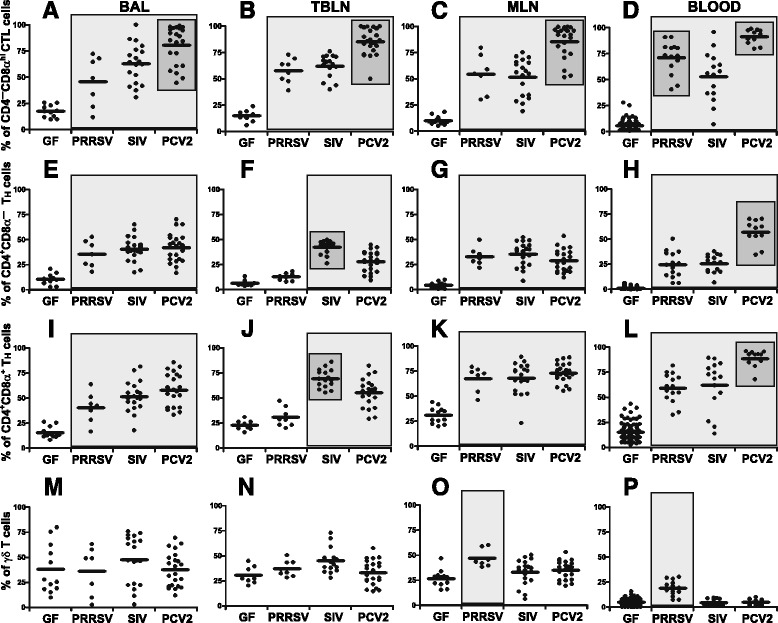
**Analysis of lymphoid cells and their subpopulations for expression of MHC-II.** Cell suspension isolated from BAL (first column), TBLN (second column), MLN (third column) and the blood (fourth column) of control GF piglets and GF piglets infected with PRRSV, SIV or PCV2 (different animal groups depicted on x-axes) were analyzed by flow cytometry for the proportion of MHC-II positive cells among selected subpopulations of T cells. Analysis included CD4^—^CD8α^hi^ CTL **(A-D)**, naive CD4^+^CD8α^—^ Th cells **(E-H)**, effector/memory CD4^+^CD8α^+^ Th cells **(I-L)** and all γδ T cells **(M-P)**. Each dot in the graphs represents an individual animal and the mean for all animals in the same group is indicated by a horizontal line. Groups of animals that show a statistically significant difference (*p* < 0.05) from GF animals are light-grey boxed and those differing even within a group are dark-grey boxed.

Unlike conventional animals where many αβ and γδ T cells express MHC-II molecules, expression of MHC-II molecules in GF piglets among individual αβ and γδ T cells is minimal (Figure 5A-P, see values for GF animals). However, all three infections caused a significant increase in the proportion of MHC-II^+^ cells among CTL (Figure 5A-D), naive Th (Figure 5E-H) and effector/memory Th cells (Figure 5I-L) in all analyzed tissues. Surprisingly, this was not seen in the TBLN of PRRSV infected piglets in which there was no increase in the expression of MHC-II among naive (Figure 5F) and effector/memory (Figure 5J) Th cells. When comparing the expression of MHC-II among αβ T cells in different infections, there was a significantly higher proportion of MHC-II^+^ cells among CTL in PCV2 infection and this was seen in all analyzed tissues (Figure 5A-D). A significantly higher proportion of MHC-II expression among Th cells was observed only in the TBLN during SIV infection (Figure 5F and J) and in the blood of PCV2 infected piglets (Figure 5H and L). It should be noted that MHC-II expression among naive Th cells is rare in conventional animals [47] and higher occurrence of MHC-II^+^CD4^+^CD8α^—^ Th cells in infected GF piglets is therefore unusual (Figure 5E-H). This phenomenon is probably related to naive immune system of GF piglets in which these cells are overrepresented as activated intermediates that further mature to CD4^+^CD8α^+^ effector/memory Th cells.

Analysis of MHC-II expression among γδ T cells revealed a significant increase only in PRRSV infection and this was observed both in the MLN (Figure 5O) and in the blood (Figure 5P).

### PCV2 infection results in a striking increase in IgA expressing cells

**Figure 6:**
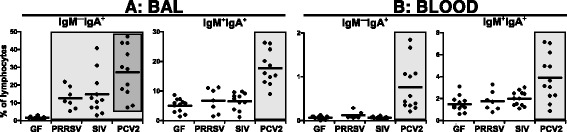
**Analysis of IgA expression on B cells.** Cell suspensions isolated from BAL **(A)** and blood **(B)** of control GF piglets and GF piglets infected with PRRSV, SIV or PCV2 (different animal groups depicted on x-axes) were analyzed by flow cytometry for the proportion of IgM^—^IgA^+^ and IgM^+^IgA^+^ cells (depicted above each graph) among gated IgM^+^ and/or IgA^+^ B cells. Each dot in the graphs represents an individual animal and the mean for all animals in the same group is indicated by a horizontal line. Groups of animals that show a statistically significant difference (*p* < 0.05) from GF animals are light-grey boxed and those differing even within a group are dark-grey boxed.

Previous studies revealed a remarkable increase in serum IgA in PCV2 infected piglets [29]. In this study we observed that the proportion of switched IgM^+^IgA^+^ and IgM^—^IgA^+^ B cells in PCV2 infection was remarkably higher in both BAL (Figure 6A) and blood (Figure 6B) than in other groups of piglets.

### PCV2 infection is characterized by a relatively high proportion of FoxP3^+^ T cells

**Figure 7:**
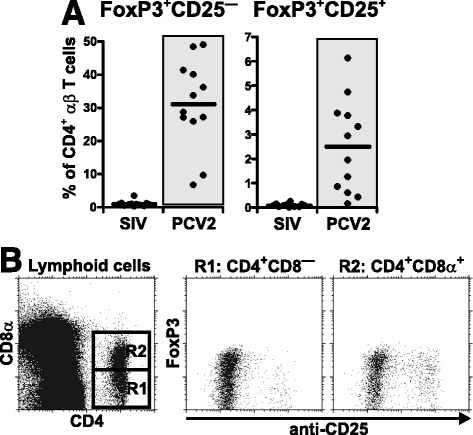
**Analysis of FoxP3** ^**+**^ **cells in SIV and PCV2 infection. (A)** Cell suspensions isolated from the blood of GF piglets infected with SIV or PCV2 (different animal groups depicted on x-axes) were analyzed by flow cytometry for the proportion of FoxP3^+^CD25^—^ and FoxP3^+^CD25^+^ cells (depicted above each graph) among gated CD4^+^ αβ T cells. Note that analysis of control GF piglets or GF piglets infected with PRRSV did not differ from SIV animals and therefore these are not shown. Each dot in the graphs represents an individual animal and the mean for all animals in the same group is indicated by a horizontal line. Groups of animals that show a statistically significant difference (*p* < 0.0001) from GF animals are light-grey boxed. **(B)** Representative detailed analysis of FoxP3 and CD25 expression by gated CD4^+^CD8α^—^ (R1) and CD4^+^CD8α^+^ (R2) αβ T cells in the blood of PCV2 infected GF piglets.

Our analysis of PRRSV, SIV and PCV2 infected animals included examination of both FoxP3 and CD25 expression by CD4^+^ αβ T cells (Figure 7). Analysis of blood lymphocytes showed that although there were no differences between PRRSV and SIV infected animals that were comparable to GF controls (data not shown), PCV2 infected animals displayed an exceptionally high proportion of FoxP3^+^ cells in both the CD25^—^ and CD25^+^ subpopulations of CD4^+^ αβ T cells (Figure 7A). These differences were so obvious that in some cases up to 50% of CD4^+^ αβ T cells expressed intracellularly FoxP3 in PCV2 animals while the number was < 2% in SIV infected animals (Figure 7A). Detailed analysis of CD4^+^CD8α^—^ and CD4^+^CD8α^+^ subpopulations of αβ T cells from PCV2 infected animals (Figure 7B) showed that while both subpopulations express FoxP3 without CD25, only effector/memory CD4^+^CD8α^+^ αβ T cells contained FoxP3^+^CD25^+^ cells.

### Diversity of B cell repertoire is non-selective after PRRSV infection but selective after SIV and PCV2 infections

**Figure 8:**
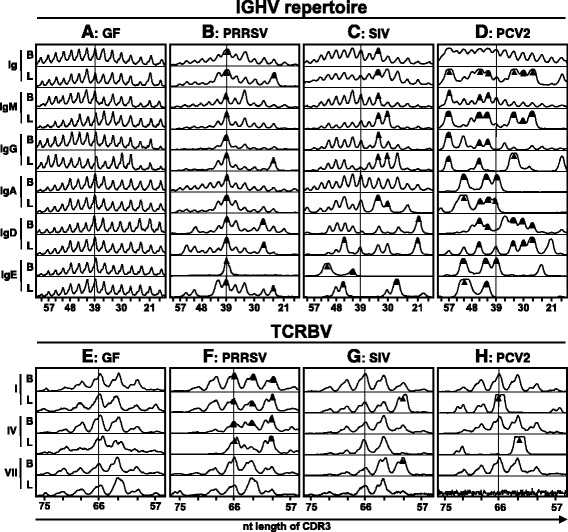
**CDR3 analysis of B (IGHV) and αβ T (TCRBV) cell receptor repertoires.** Representative CDR3 length analysis (spectratyping) of total cells isolated from the blood and BAL (indicated next to spectrotyping curves by “B” and “L” respectively) of control GF piglets **(A, E)** and GF piglets infected with PRRSV **(B, F)**, SIV **(C, G)** or PCV2 **(D, H)**. Analysis of IGHV repertoire **(A-D)** was done for total immunoglobulins (Ig) and also for IgM, IgG, IgA, IgD and IgE classes. Analysis of TCRBV repertoire **(E-H)** included amplification for VβI - VβIII families (I), VβIV - VβVI families (IV) and VβVII family (VII). For both IGHV and TCRBV, shared bands or bands of interest are indicated by a dark triangle. Vertical lines indicate the mean CDR3 length in the non-selected repertoire of GF piglets. Lengths of CDR3 are indicated on x-axes and include number of nucleotides (nt) from the 3′ end of V segment to the 3′ end of J segment. The results are representative of three independent experiments.

We tested whether there was preferential expansion of certain B cell clones during the different viral infections by IGHV-CDR3 spectratyping of total VDJ_H_ rearrangements associated with various Ig isotypes recovered from total cDNA prepared from the blood and BAL. The spectratypic analysis indicated a Gaussian and unselected profile in GF animals for all isotypes (Figure 8A). PRRSV infection (Figure 8B) was characterized by selective expansion of clones with a length near the center of the Gaussian distribution of IGHV-CDR3 lengths (~39 nt). These were emphasized in total Ig and shared among all immunoglobulin classes in the both blood and BAL as previously reported [6]. Several less prominent and smaller bands (~24-27 nt) were also found for IgG, IgD and IgE especially in the BAL (Figure 8B). The overall profile suggests a non-antigen selective expansion of the most frequent B cell clones from the pre-immune repertoire in all Ig classes. Since the same pattern is seen in the blood and BAL, it is likely that the same clones are expanded and/or distributed to many other tissues. By contrast, analysis of IGHV-CDR3 spectratype in SIV infected animals (Figure 8C) revealed the expansion of certain clones with a length out of Gaussian distribution center (~33 nt). These were shared among IgM and IgG in the blood and IgM, IgG and IgA in the BAL. Distinctly separate IgD and IgE clones of different length were expanded. This SIV profile is typical of an antigen-specific and isotype-associated proliferation of B cell clones. The profile in PCV2 infection (Figure 8D) is also in stark contrast to what is seen in PRRSV infection (Figure 8B). The PCV2 profile is also characteristic of antigen-specific clonal expansion with many clones falling outside the Gaussian distribution center. Some were shared by IgM, IgG and IgD mainly in the BAL while others with different length were shared by IgA and IgE in the BAL as well as in the blood (Figure 8D). The latter is a relevant observation because of the remarkable increase of switched IgM^+^IgA^+^ and IgM^—^IgA^+^ B cells in PCV2 infection (Figure 6). Unfortunately, there is no useful mAb against IgE in swine to allow us to test whether switched IgE^+^ B cells are as frequent as IgA^+^ in PCV2 infection. The PCV2 profile also suggests that piglets may recognize more viral epitopes than do piglets infected with SIV.

### Diversity of TCRαβ repertoire is polyclonal after PRRSV infection but selective after SIV and PCV2 infections

Clonal diversity of αβ T cells was measured by TCRBV-CDR3 spectratyping using three sets of primers for three Vβ superfamilies (VβI - VβIII, VβIV - VβVI and VβVII). Thus, all seven Vβ gene families known in swine were targeted. In GF piglets, the pattern was more or less polyclonal for all Vβ families (Figure 8E). In PRRSV infected piglets, T cell clones expressing members of the most Vβ families (VβI-VβVI) were prominently expanded and were found in the blood as well as BAL (Figure 8F). This contrasted with both SIV and PCV2 infected piglets in which only αβ T cells expressing certain CDR3 length were selectively expanded (Figure 8G and 8H respectively). Collectively, these observations suggest antigen-specific T cell stimulation in the respiratory tract of SIV and PCV2 infected piglets but systemic and polyclonal activation of αβ T cells in the case of PRRSV.

## Discussion

The absence of complex information on lymphocyte subsets in two of the most persistent and pandemic viral infections of newborn piglets was the basis for the studies undertaken. SIV was used as a reference viral infection since it is resolved even by GF piglets by well-established mechanisms [4,5]. Using this approach we hoped to gain insight into why PRRSV and PCV2 infections are not readily resolved like SIV. The comparison is not ideal since SIV targets epithelial cells [4] while both PRRSV [9] and PCV2 [14] target cells of the immune system, and PRRSV [9,14] and SIV [4] are RNA viruses while PCV2 is a small DNA virus [3,13,14]. Given this restriction, we reasoned that by using isolator piglets, the more direct effect of these infections on the piglet immune system could be realized.

Our previous studies suggest that PRRSV causes dysregulation of the B cell compartment resulting in hypergammaglobulinemia and polyclonal B cell activation [28]. Later, we showed that polyclonal expansion of the pre-immune repertoire shows little evidence of diversification [6,42]. A similar pattern is seen with piglets experimentally infected in utero [7]. The phenotype of the B cells temporally-associated with this polyclonal responses is unknown. We show here for the first time that PRRSV infection caused a severe reduction of primed CD2^—^CD21^+^ B cells and an extreme increase in effector CD2^+^CD21^—^ AFC/PC cells. Since primed CD2^—^CD21^+^ B cells normally arise from naive CD2^+^CD21^+^ B cells [24,26], our observation suggests that PRRSV circumvents normal priming pathway and causes non-specific activation and rapid differentiation of naive B cells into AFC/PC which by-pass repertoire diversification. The mechanism of this dysregulation could be that PRRSV: (a) interacts directly with naive B cells causing their non-specific activation thereby omitting the specific priming step, (b) activates non-specifically Th cells that engage naive B cells resulting in the accelerated activation pathway so that primed B cells do not accumulate and therefore appear depleted or (c) manipulates Mo/MF/DC that in turn influences B and Th cells in a similar way as described above. Whatever the mechanism, immunoglobulins secreted by the high proportion of effector CD2^+^CD21^—^ AFC/PC cells and their resting CD2^—^CD21^—^ AFC/PC progenies in the blood may explain the observed hypergammaglobulinemia in all immunoglobulin classes that characterize this infection in isolator and fetal piglets [6,7,28] and also the robust production of Abs in conventional animals [9,10]. It might also explain why < 1% of serum Igs are virus specific [28] and Abs are non-virus neutralizing [10] because they are derived by expansion of the non-selected B cell repertoire and not through germinal center formation, selection and affinity maturation [6]. The possibility that polyclonal B cell differentiation by-passes germinal center formation agrees with the findings that considerable changes for B cells and their subpopulations observed in the BAL were not reflected in the TBLN.

PRRSV infects and replicates in Mo/MF/DC and impairs their normal antigen presentation, distorts the cytokine profile and diminishes the expression of costimulatory molecules [48]. We therefore prefer the explanation that virus manipulates Mo/MF/DC that in turns influences Th cells and these thereafter B cells. Here we show that PRRSV elevates the proportion of effector/memory CD4^+^CD8α^+^ Th cells and cause polyclonal activation of members of nearly all Vβ families as documented by TCRVB-CDR3 spectratyping. Evidence that Th cells are activated non-specifically outside of germinal centers agrees with the finding that both subsets of Th cells in the TBLN of PRRSV infected piglets are mostly MHC-II^—^ and therefore similar to those in GF controls. If the activation of Th cells would have taken place in TBLN, they should express MHC-II as a result of previous activation [47]. Most probably, non-specific activation of B cells occurs just at the site of infection because BAL is the only tissue where reduction of primed B cells was observed and where the highest proportion of effector AFC/PC was recorded. Moreover, the B cell spectratype shown in Figure 8B and the more extensive clonal analyses previously published [6] do not document simple expansion of all B cell clones but rather selective expansion of the same B cell clones distributed to many tissues. These might be B cell clones that can interact with the polyclonally-activated Th cells. If there would be direct effect of PRRSV on B cells or Mo/MF/DC only, the profile of IGHV-CDR3 spectratype should be polyclonal and not selective. In this respect, the effect of PRRSV on B cell activation somewhat resembles lymphocytic choriomeningitis virus (LCMV) infection in which specific Th cells recognize and directly activate B cells that have processed viral antigens irrespective of the B cell receptor specificity [49]. Involvement Mo/MF/DC and their effect on T cells is also documented by observation that highly pathogenic PRRSV causes thymic atrophy, presumably by activation of CD4^+^CD8^+^ T cell precursors in thymic cortex that subsequently undergo apoptosis and are eliminated [50]. In any case, a role of Mo/MF/DC, Th and B cells in persistence of PRRSV must be confirmed in conventional animals with regard also to other factors that include a complex viral structure, redirection of the humoral response towards non-surface proteins, antigenic and genetic drifts and subversion of interferon gene induction [10].

PRRSV infection was also characterized by an increase in the proportion of CD2^+^CD8α^+^ γδ subset in all tissues. While the role of CD2^+^CD8α^+^ γδ T cells is still unclear, it is the only subset of γδ T cells possessing cytotoxic activity [51]. Recent studies show that these cells represent a terminally differentiated subset [27] that arises from other CD2^+^ γδ T cells upon activation [30]. An involvement of CD8^+^ γδ T cells in PRRSV infection was reported earlier [52] and these cells were shown to produce IFN-γ [12]. Thus, CD2^+^CD8α^+^ γδ T cells may facilitate clearance of PRRSV even when other mechanisms are dampened. This can be especially relevant for PRRSV infection where Abs are superfluous and viremia may be resolved without detectable levels of neutralizing Abs [11].

Fatal PMWS caused by PCV2 infection may develop in GF animals, and it is associated with generalized lymphopenia [8]. In this study, we observed this outcome in only one GF animal that was inoculated with the PCV2 688 isolate (Additional file 2) while other investigators report 40% mortality in SPF animals [17] and ~50% of conventional animals [18]. The extent of PCV2 viremia may also differ among GF piglets [8], colostrum-deprived piglets [16], and conventional pigs [1]. Clinical PMWS, unlike SIV and PRRSV, is often late to develop and occurs beyond the 5-6 weeks in which GF piglets can be maintained in isolators. Based on our experience with the PCV2 688 isolate, PMWS resulting in lymphopenia and subsequent death is rare among GF piglets, suggesting that PMWS results from opportunistic pathogens or other factors that modulate the immune system. On the other hand, lymphopenia itself should not be lethal in GF animals that are free of any pathogens. Therefore, other intrinsic factors of mortality need to be considered.

While symptoms of PMWS in our piglets were rare, the prevalence of CTL was striking and is consistent with reports by others [18,21]. An exceptionally high proportion of CTL with MHC-II^+^ phenotype was found in all organs. The role of elevated CTL in PCV2 infection is unclear. This increase may be connected to higher cytotoxic activity against PCV2 infected cells. However, the slow replication rate of PCV2 and its persistent nature [14] questions the effectiveness of such CTL. The expansion of CTL has been associated with an experimental autoimmune syndrome that destroys lymphocytes when one type of virus is challenged by another virus but not vice versa [53]. This resembles pigs inoculated with PRRSV before PCV2 but not vice versa [54]. It is therefore possible that PCV2 may expand potentially autoimmune CTL that under certain circumstances facilitate destruction of lymphocytes, which is a hallmark of PCV2 infection [16–18]. In that case, the development of lymphopenia should be strongly linked to MHC-I [53]. In support of this notion, host genetic differences in susceptibility to PCV2 associated lymphoid lesions were noted among different breeds of pigs [55]. In any case, we have currently no evidence of autoimmunity in PCV2 infected piglets. Therefore, the speculation about the role of autoimmune CTL must be carefully verified together with exclusions of other potential explanations of PCV2 pathogenesis, such as infection and destruction of lymphocyte precursors, replication capacity of PCV2 in actively dividing/resting lymphocytes and/or viral-induced or cytokine-induced apoptosis [3].

The increase in the proportion of CTL in PCV2 infection was associated with a decrease in the proportion of both naive CD4^+^CD8α^—^ and effector/memory CD4^+^CD8α^+^ Th cells in the respiratory tract. Interestingly, this restriction was not observed in the blood where Th cells also displayed highest MHC-II expression. Further analysis revealed that many of these Th cells in the blood express FoxP3 in both CD25^—^ and CD25^+^ fraction. While CD25^+^CD4^+^FoxP3^+^ probably represent T regulatory cells [56], the role of CD25^—^CD4^+^FoxP3^+^ T cells has to be elucidated. T regulatory cells would not be expected to cause lymphopenia, which is supported by the general wellness of our infected GF piglets. However, immunosuppression may compromise protective immune responses to secondary pathogens, known to be a feature of porcine circovirus-associated diseases [14,21].

B cell frequency and subpopulation usage in PCV2 infections is comparable to SIV. Presumably this explains why asymptomatic animals can mount an effective humoral response and eventually clear the virus [16,20]. This may also explain the partial effectiveness of PCV2 vaccines [3]. However, when PCV2 infection leads to PMWS and associated lymphopenia, the B cell compartment is destroyed and effective humoral responses fail to occur [16].

An interesting feature of PCV2 infected GF piglets was the pronounced expansion of certain IgA and IgE clones that mostly appear distinct from IgM, IgG or IgD clones (Figure 8H). This is suggestive of an antigen-specific selection in the lower respiratory tract, i.e. at the site of infection. It is well known that IgA B cells stimulated at one mucosal site traffic through the blood to other sites [57], and that IgA produced in the TBLN is a major contributor to circulating IgA [58]. This could explain the surprisingly high levels of serum IgA in PCV2 infected piglets that on average are 10 fold higher than in SIV infected piglets [29]. However, IgA levels are variable [29] which agrees with the variability in the amount of switched IgM^+^IgA^+^ and IgM^—^IgA^+^ B cells among animals (Figure 6). Thus, local antigen-specific IgA may play a more important role in controlling the severity of PMWS in contrast to SIV in which IgG levels in the BAL are 3–6 fold higher than for IgA [5,29]. This may reflect the fact that PCV2 is not exclusively a respiratory virus [3,13]. It would be wise to determine, using conventional or SPF piglets, whether a correlation exists between virus-specific IgA and prevention of PMWS. The role of the profoundly expanded IgE clones in PCV2 infection is more difficult to explain, although some studies indicate that some viruses act like parasites and elevate IgE response [59]. In all of these cases there is a need to determine if elevation of a particular isotype parallels the increase in specific Abs.

The study reported has several weaknesses. First, the expense and determinant nature of isolator piglet studies made it initially impractical to conduct kinetic studies using lymphocytes collected from different tissue throughout the study, this could only be done for blood. Second, isolator piglets can only be held for 5–6 weeks, limiting our observation to this window of development. Nevertheless, by comparison to data from blood which was kinetically collected, our data show that the most pronounced changes in lymphocyte subsets of interest occurred at the time of sacrifice for both PRRSV and PCV2 infected isolator piglets. Therefore it is unlikely that major changes that occurred before the time of sacrifice differed from what we observed at sacrifice. Third, comparing the absolute and relative data for blood shows that there can be merit to measure both of these values. Naturally, applying this practice to individual lymphoid tissues is a major technical undertaking. However, it could provide further insight into the immunobiology of PRRS. Another shortcoming of the data we present is that it does not correlate the changes in lymphocyte subsets to their antigen specificity. Before such studies are undertaken, some universal agreement is needed as to regard which antigens should be studied. When a consensus is reached, our data can be useful in selecting what lymphocyte subsets should be studied.

In summary, this is the first truly comparative study on the direct effect of three important viral infections of piglets on lymphocyte populations in a highly controlled experimental model. Despite some weakness, we believe our studies provide the best data reported so far on the effect of important viral diseases on lymphocyte populations in a setting in which the results cannot be affected by other environmental factors. Our findings are not a magic bullet for the resolution of these viral infections. However, combined with our previous humoral studies [5–7,29], our observations now make it possible to propose testable hypotheses that can address the mechanism of immune dysregulation by PCV2 and PRRSV. For example, is germinal center and AID expression normal or retarded in PRRSV? Are early anti-PRRSV Abs of low affinity because germinal center formation is bypassed? What viral factors are responsible for the reduction of primed B cells during PRRSV infection? Can depletion of CD2^+^CD8α^+^ γδ T increase the pathogenicity of PRRSV infection? Are CTL involved in depletion of lymphocytes during PMWS? Can sequential infection with PCV2 recruit autoimmune CTL or cause CTL exhaustion in infected animals with different viruses or different PCV2 isolates? Is there a correlation between virus-specific IgA and prevention of PMWS? Can mucosal vaccines and mucosal adjuvants result in a more efficacious PCV2 vaccine? What is an outcome of PCV2 infection in inbred pigs? Does elevated level of FoxP3^+^ cells in PCV2 infected animals affect the effectiveness of PCV2 vaccines, the response of piglets to secondary infections or the activities of other leukocyte populations? Addressing these questions might guide efforts to prove or develop the next generation of vaccines for two of the most important viral infections of young piglets.

## Additional files

Additional file 1:
**Gating strategy for flow cytometry analysis of individual lymphoid subsets.** Representative figure showing the gating strategy and analyzed lymphoid subsets used in this study **(A-K)**. Note that gate used for each analysis is depicted above individual dotplots and analyzed populations are pointed by doted lines. Combinations of antibodies used are depicted in the lowest part of figure **(M)** and their usage for final analysis is depicted above individual dotplots in circled numbers. See Materials and methods for detailed description. Additional file 2:
**Flow cytometry analysis of asymptomatic and PMWS symptomatic GF piglets infected with PCV2.** Cell suspensions isolated from the blood **(A-F)** and BAL **(G-L)** of GF piglets infected with PCV2 were analyzed by flow cytometry for the proportion of lymphoid cells in region R1 (first columns), CD2^+^ cells and B cells (second columns) and αβ / γδ T cells (third columns). One representative PCV2 infected piglet that did not show clinical signs of PMWS **(A-C and G-I)** and one animal that showed clinical signs of PMWS and died **(D-F and J-L)** are shown. Note that there is marked lymphopenia of B cells **(E and K)** and T cells **(F and L)** in symptomatic PCV2 infected piglet. Additional file 3:
**Leukograms for individual infections.** Leukograms showing number of white blood cells (first column), neutrophils (second column), lymphocytes (third column) and monocytes (fourth column) in the blood of SIV (first row), PRRSV (second row) and PCV2 (third row) infected animals during course of infection (x-axes) in comparison with control GF piglets. Control GF piglets are depicted in black while infected GF piglets are in red. Each line represents one animal. Note that leukograms were not performed on all animals. The level of statistical significance is depicted above data points for each dpi (ns = not significant). Additional file 4:
**Analysis of recalculated absolute numbers of lymphoid cells and their subpopulations in the blood.** Relative data from the blood of control GF piglets and GF piglets infected with PRRSV, SIV or PCV2 (different animal groups depicted on x-axes) were recalculated based on leukogram results for the absolute numbers of B cells **(A)** and their subpopulations **(E-H)**, αβ T cells **(B)** and their subpopulations **(I-K)**, γδ T cells **(C)** and their subpopulations **(L-N)** and NK cells **(D).** The phenotype of individual cell populations is depicted above each graph. Bars represent mean values obtained during monitoring of animals at dpi 0 (open bars), 7 (light-grey bars), 14 (dark-grey bars), 21 (light-black bars) and 28 (black bars). Error bars represent ± standard deviation. Values that show statistically significant difference (*p* < 0.05) from day 0 values are indicated by asterisk.

## Abbreviations

AFC/PC: Antigen forming cells and plasma cells
BAL: Bronchoalveolar lavage
CCID_50_: Cell concentration at infection for 50% tissue-culture infectious dose
FoxP3: Forkhead box P3 protein
FSC/SSC: Forward scatter versus side scatter
GF: Germ-free
IGHV: Immunoglobulin heavy chain variable region
MLN: Mesenteric lymph node
Mo/MF/DC: Monocyte/macrophage/dendritic lineage cells
PBS-GEL: PBS containing 0.1% sodium azide and 0.2% gelatin from cold water fish skin
PCV2: Porcine circovirus type 2
PMWS: Postweaning multisystemic wasting syndrome
PRRSV: Porcine reproductive and respiratory syndrome virus
SIV: Swine influenza virus
TCRBV: TCRβ variable region
TBLN: Tracheobronchial lymph node
