# Supplementary material for: The comparative profile of lymphoid cells and the T and B cell spectratype of germ-free piglets infected with viruses SIV, PRRSV or PCV2
Source: Vet Res. 2014 Sep 4;45(1):91. doi: 10.1186/s13567-014-0091-x (PMC4156959; doi:10.1186/s13567-014-0091-x)
Supplement: Additional file 1: — Gating strategy for flow cytometry analysis of individual lymphoid subsets. Representative figure showing the gating strategy and analyzed lymphoid subsets used in this study (A-K). Note that gate used for each analysis is depicted above individual dotplots and analyzed populations are pointed by doted lines. Combinations of antibodies used are depicted in the lowest part of figure (M) and their usage for final analysis is depicted above individual dotplots in circled numbers. See Materials and methods for detailed description. [file 13567_2014_91_MOESM1_ESM.pdf]

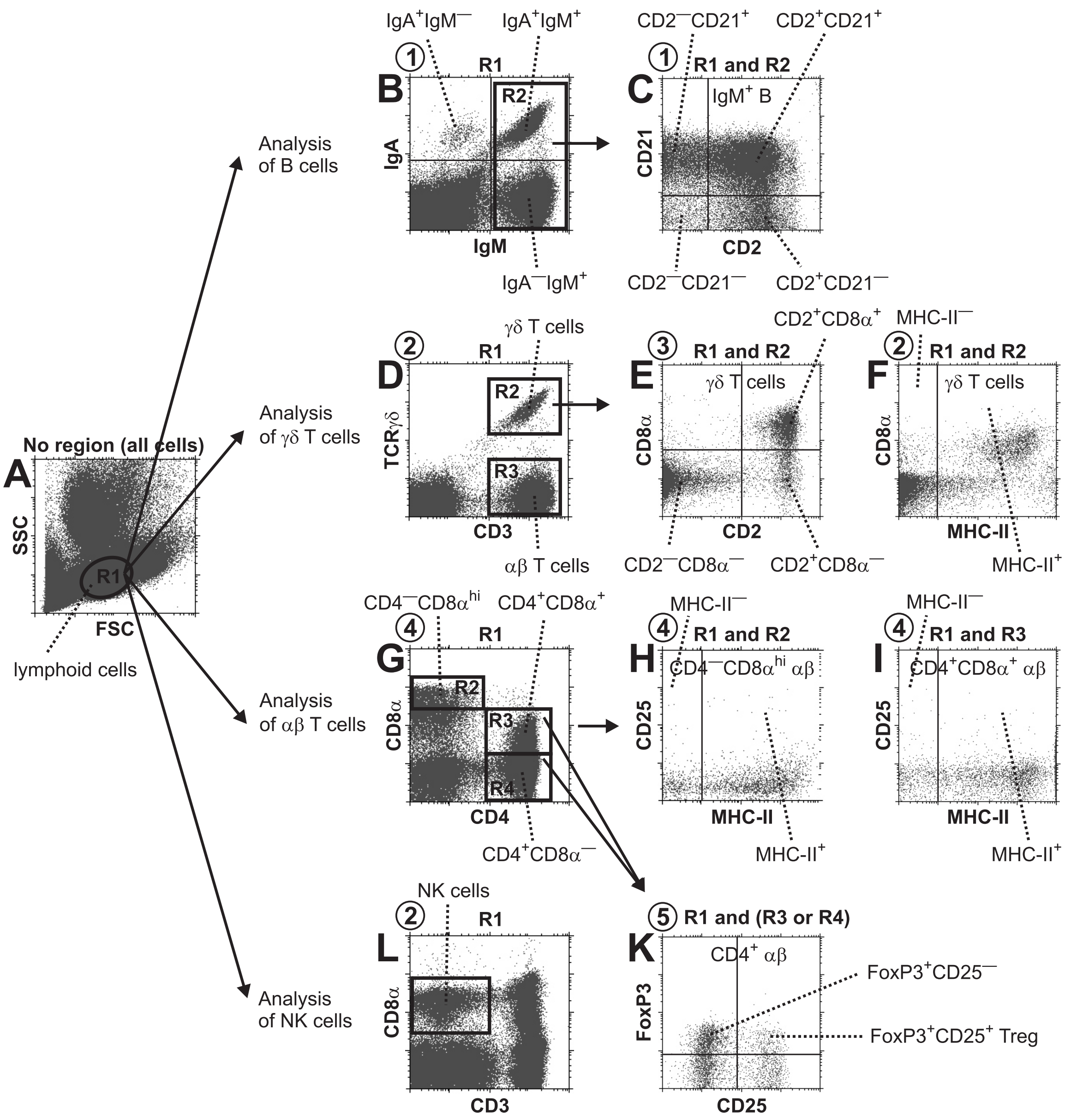

M

Staining 1 - version A:
anti-IgM (M160)
anti-CD2 (1038H-5-37)
antiCD21 (IAH-CC51)
anti-IgA (M1456)

Staining 2 - version A:
anti-CD3 (PPT3)
anti-MHC-II (1038H-12-34)
anti-TCRγδ (PPT16)
anti-CD8α (76-2-11)

Staining 3 - version A:
anti-CD8α (76-2-11)
anti-CD2 (1038H-5-37)
anti-TCRγδ (PPT16)
anti-CD25 (K231-3B2)

Staining 4 - version A:
anti-CD8α (76-2-11)
anti-MHC-II (1038H-12-34)
anti-CD4 (10.2H2)
anti-CD25 (K231-3B2)

Staining 5:
CD25 (K231-3B2)
CD4 (10.2H2)
FoxP3-biotin (FJK-16s)
CD8α (76-2-11)

Staining 1 - version B:
anti-IgM (M160)
anti-CD2 (1038H-5-37)
antiCD21-biotin (IAH-CC51)
anti-IgA (M1456)

Staining 2 - version B:
anti-CD3 (PPT3)
anti-MHC-II (1038H-12-34)
anti-TCRγδ-biotin (PPT16)
anti-CD8α (76-2-11)

Staining 3 - version B:
anti-TCRγδ (PPT16)
anti-CD2 (1038H-5-37)
anti-CD8α-biotin (76-2-11)
anti-CD25 (K231-3B2)

Staining 4 - version B:
anti-CD8α (76-2-11)
anti-MHC-II (1038H-12-34)
anti-CD4-biotin (10.2H2)
anti-CD25 (K231-3B2)
