# Supplementary figures and images for: The comparative profile of lymphoid cells and the T and B cell spectratype of germ-free piglets infected with viruses SIV, PRRSV or PCV2
Source: Vet Res. 2014 Sep 4;45(1):91. doi: 10.1186/s13567-014-0091-x (PMC4156959; doi:10.1186/s13567-014-0091-x)

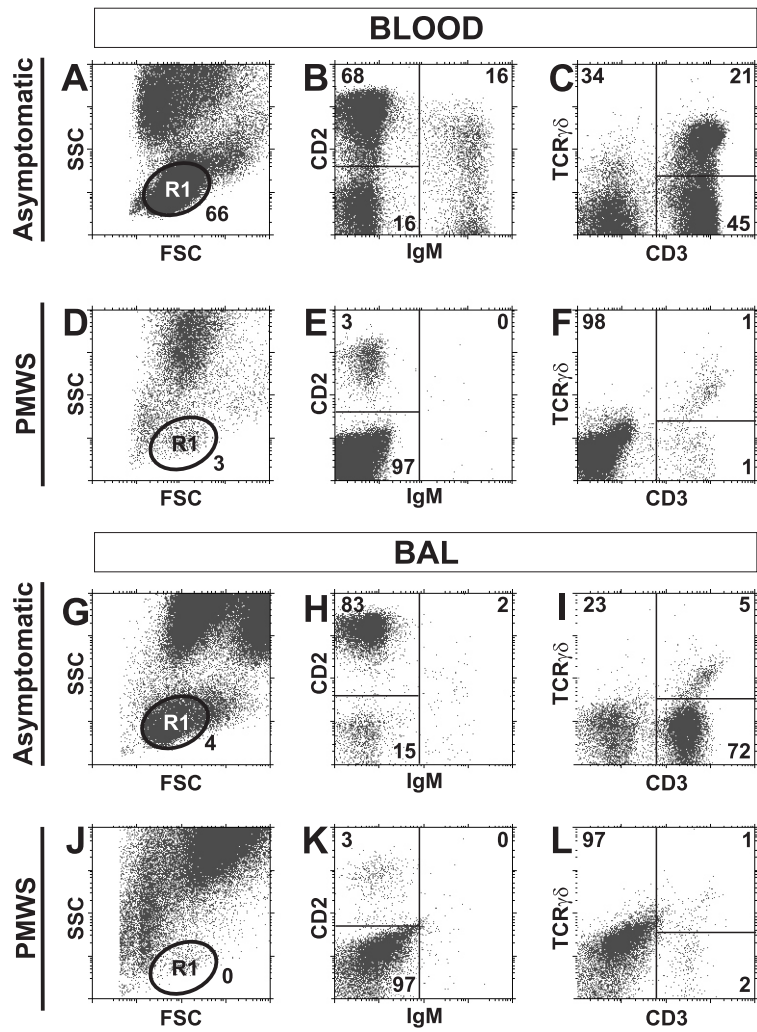

Supplement: Additional file 2: — Flow cytometry analysis of asymptomatic and PMWS symptomatic GF piglets infected with PCV2. Cell suspensions isolated from the blood (A-F) and BAL (G-L) of GF piglets infected with PCV2 were analyzed by flow cytometry for the proportion of lymphoid cells in region R1 (first columns), CD2+ cells and B cells (second columns) and αβ / γδ T cells (third columns). One representative PCV2 infected piglet that did not show clinical signs of PMWS (A-C and G-I) and one animal that showed clinical signs of PMWS and died (D-F and J-L) are shown. Note that there is marked lymphopenia of B cells (E and K) and T cells (F and L) in symptomatic PCV2 infected piglet. [file 13567_2014_91_MOESM2_ESM.pdf]

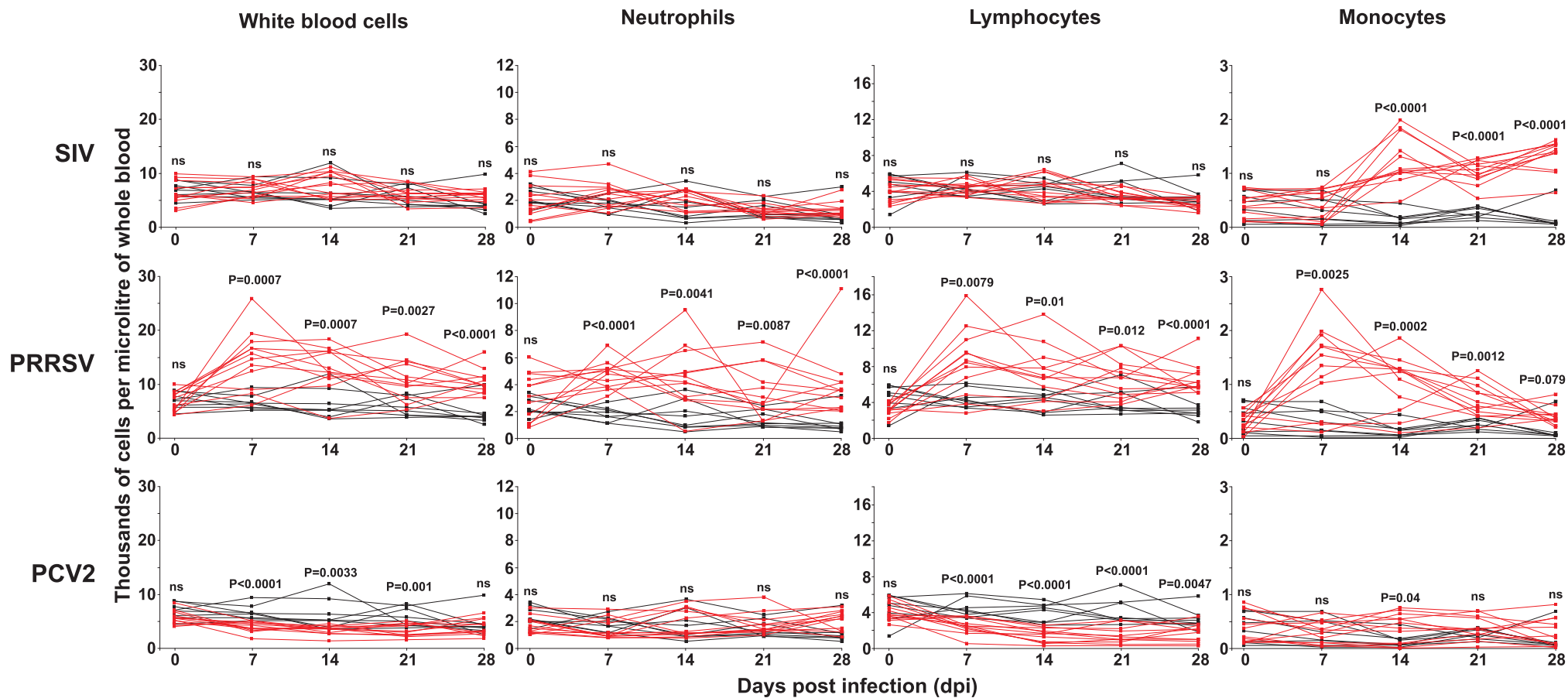

Supplement: Additional file 3: — Leukograms for individual infections. Leukograms showing number of white blood cells (first column), neutrophils (second column), lymphocytes (third column) and monocytes (fourth column) in the blood of SIV (first row), PRRSV (second row) and PCV2 (third row) infected animals during course of infection (x-axes) in comparison with control GF piglets. Control GF piglets are depicted in black while infected GF piglets are in red. Each line represents one animal. Note that leukograms were not performed on all animals. The level of statistical significance is depicted above data points for each dpi (ns = not significant). [file 13567_2014_91_MOESM3_ESM.pdf]

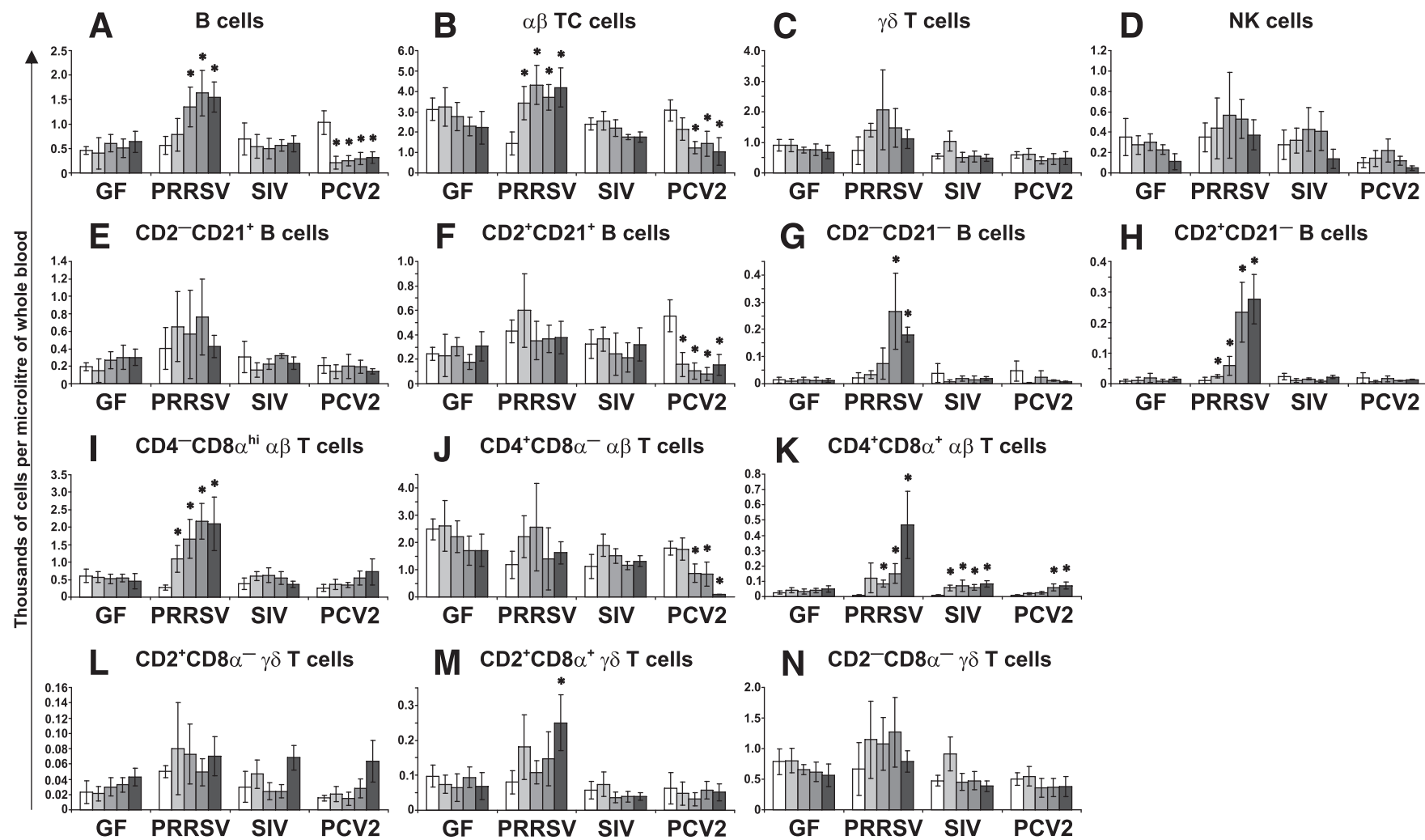

Supplement: Additional file 4: — Analysis of recalculated absolute numbers of lymphoid cells and their subpopulations in the blood. Relative data from the blood of control GF piglets and GF piglets infected with PRRSV, SIV or PCV2 (different animal groups depicted on x-axes) were recalculated based on leukogram results for the absolute numbers of B cells (A) and their subpopulations (E-H), αβ T cells (B) and their subpopulations (I-K), γδ T cells (C) and their subpopulations (L-N) and NK cells (D). The phenotype of individual cell populations is depicted above each graph. Bars represent mean values obtained during monitoring of animals at dpi 0 (open bars), 7 (light-grey bars), 14 (dark-grey bars), 21 (light-black bars) and 28 (black bars). Error bars represent ± standard deviation. Values that show statistically significant difference (p < 0.05) from day 0 values are indicated by asterisk. [file 13567_2014_91_MOESM4_ESM.pdf]
